# Phase relations of interneuronal activity relative to theta rhythm

**DOI:** 10.3389/fncir.2023.1198573

**Published:** 2023-07-06

**Authors:** Ivan Mysin

**Affiliations:** Laboratory of Systemic Organization of Neurons, Institute of Theoretical and Experimental Biophysics of Russian Academy of Sciences, Pushchino, Russia

**Keywords:** CA1 field, short-term plasticity, conductance-based refractory density approach, gradient descent, leaky integrate-and-fire (LIF) model

## Abstract

The theta rhythm plays a crucial role in synchronizing neural activity during attention and memory processes. However, the mechanisms behind the formation of neural activity during theta rhythm generation remain unknown. To address this, we propose a mathematical model that explains the distribution of interneurons in the CA1 field during the theta rhythm phase. Our model consists of a network of seven types of interneurons in the CA1 field that receive inputs from the CA3 field, entorhinal cortex, and local pyramidal neurons in the CA1 field. By adjusting the parameters of the connections in the model. We demonstrate that it is possible to replicate the experimentally observed phase relations between interneurons and the theta rhythm. Our model predicts that populations of interneurons receive unimodal excitation and inhibition with coinciding peaks, and that excitation dominates to determine the firing dynamics of interneurons.

## 1. Introduction

The hippocampus is a brain structure that plays a key role in the processes of attention and memory. To process information, neural ensembles in the hippocampus need to be synchronized with rhythms. The main rhythm that organizes the neural activity of the hippocampus during cognitive tasks is the theta rhythm (4–12 Hz) (Vinogradova, 1995; Buzsáki, 2002; Buzsáki and Moser, 2013). Almost all hippocampal neurons are modulated by theta rhythm (Mizuseki et al., 2009). This is expressed in the fact that each population has a theta rhythm phase, in which the probability of discharges of its neurons is maximal. Modulation of neuronal activity by rhythm makes it possible to synchronize different areas of the hippocampal formation during information processing (Fries, 2015; Nuñez and Buño, 2021; Mysin and Shubina, 2022). In addition, the theta rhythm provides an ordered structure of the place cell activity due to phase precession (Burgess and O'Keefe, 2011; Jaramillo and Kempter, 2017). Thus, understanding the mechanisms of theta rhythm formation is the most important problem of neuroscience.

Our study has two aims. The first aim is to explain the effect of modulation of the firing rate of the interneurons of the CA1 field by the theta rhythm. The second aim is to adapt the optimization methods of spike network models to describe experimental data.

The mechanisms of formation of phase relations of interneuronal activity and theta rhythm are unknown. Several theoretical studies have investigated the formation of phase relations between neurons of the CA1 field and theta rhythm (Bezaire et al., 2016; Mysin, 2021). Despite the detailed nature of these models, the authors were unable to reproduce the form of distribution of most types of interneurons in the theta rhythm phase. The main problem in constructing a model of the distribution of neurons by theta rhythm phases is to identify the mechanism of stabilization of antagonistic relationships between different populations of interneurons. For example, parvalbumin-containing (PV) and cholecystokinin-containing (CCK) basket neurons inhibit each other and fire at opposite phases of the theta rhythm (Lasztóczi et al., 2011; Royer et al., 2012; Varga et al., 2012; Bezaire and Soltesz, 2013; Somogyi et al., 2014). It is difficult to choose the parameters of connections when these neurons form a stable antiphase activity. Most often, one of the populations completely inhibit the other. When considering a larger number of interneuron populations, the problem becomes much more complicated (Bezaire et al., 2016; Mysin, 2021).

We hypothesized that short-term synaptic plasticity stabilizes the structure of interneuronal activity. In a recent study, the authors of the project Hippocampome.org provide a large meta-analysis of data on short-term synaptic plasticity in the hippocampus (Moradi et al., 2022). We used the estimates of this study as the basis of our model. We have considered the mechanism of formation of phase relations for populations of interneurons of the CA1 field. The CA1 field was chosen because there is the greatest amount of data on interneuron activity for this region of the hippocampus. We modeled seven populations of interneurons: PV (pvbas) and CCK (cckbas) basket, axo-axonal (aac), OLM (oriens-lacunosum moleculare) (olm), Ivy (ivy), and neurogliaform (ngf) and bistratified (bis) neurons. These populations were selected because the phase relationships for them are described (Somogyi and Klausberger, 2005; Fuentealba et al., 2010; Lapray et al., 2012; Varga et al., 2012). Neurons in these populations make up ~70% of all interneurons in the CA1 field (Bezaire and Soltesz, 2013). We also took into account inputs from pyramidal neurons of the CA3 and CA1 fields, as well as from neurons of the third layer of the entorhinal cortex. We were able to select the parameters of neurons and connections in the model and found a good description of the mechanisms of phase relations.

Considering a network of seven populations requires setting up several hundred parameters. This makes it impossible to apply gradient-free optimization methods. We were able to adapt the model to apply gradient descent optimization. This approach can be applied to modeling problems of other experimental phenomena in neural networks of the brain.

## 2. Materials and methods

### 2.1. Models of neurons

**Figure 1:**
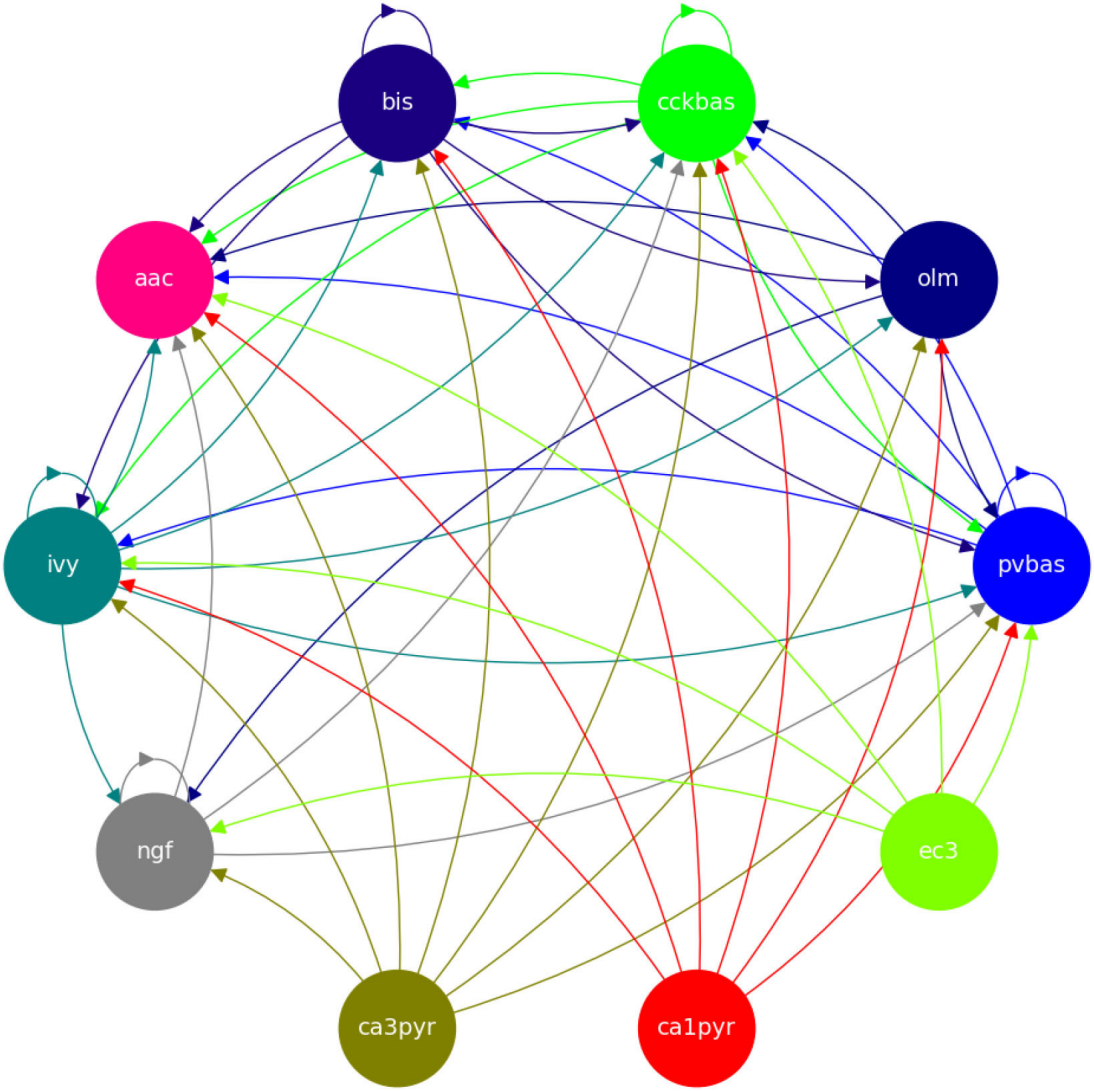
Model architecture. Hippocampal CA1 microcircuit is simulated. Cell types and their connectivity are shown. ca3pyr, pyramidal neurons of the CA3 field; ca1pyr, pyramidal neurons of the CA1 field; ec3, principal neurons of the 3 layer of the entorhinal cortex; pvbas, PV basket cells; olm, Oriens-lacunosum molecular cells; cckbas, CCK basket cells; ivy, Ivy cells; ngf, neurogliaform cells; bis, bistratified cells; aac, axo-axonal cells. Neurons of the third layer of the entorhinal cortex and pyramidal neurons of the CA3 and CA1 fields are not simulated explicitly. They were taken into account as external inputs to the model. All interneurons are simulated with the LIF model.

We consider a network of seven populations (Figure 1). The neurons of each population are described by the leaky integrate-and-fire (LIF) model. We used simulations using the conduction-based refractory density (CBRD) approach (Section 2.3) and direct stimulation of neurons with noise input (Monte Carlo). In this section, we will only describe the equations used. In Section 3.1, we will discuss in detail the motivation for using each approach for modeling. Monte Carlo simulations were provided with equations as follows:


(1) $$\begin{array}{l} C_{m}\frac{dV}{dt}=g_{L}\left(E_{L}-V\right)+I_{ext}+\sum I_{syn}+\sigma_{m}\cdot \eta \left(t\right)\\ \text{if}\;V>V^{T}:\;V=V_{reset} \end{array}$$

Here, *C*_*m*_ is the membrane capacitance, *V* is the membrane potential, *g*_*L*_ is the leak conductance, *E*_*L*_ is the leak reverse potential, *I*_*ext*_ is the external current, and *I*_*syn*_ is the synaptic current. σ_*m*_ is the standard deviation of noise η~N(0,1). For all neurons in all simulations, we used following values: $C_{m}=1\;\mu F/cm^{2},\;g_{L}=0.1\;mS/cm^{2},\;E_{L}=-60\;mV,\;V^{T}=-50\;mV,\;V_{reset}=-90\;mV,\;\sigma_{m}=0.3\;\mu A/cm^{2}/\sqrt{ms}$. Value *I*_*ext*_ is optimized for each population. After generating the action potential, the refractory = 3 *ms*. We used typical values used in simulations. The exact values of the parameters can be viewed in the Hippocampome database (Venkadesh et al., 2019)

Population frequency for Monte Carlo simulations as follows:


(2) $$\begin{array}{l} \nu =\frac{n_{fired}}{N\cdot \text{Δ}t} \end{array}$$

where *n*_*fired*_ is the number of fired cell at each time step, *N* = 4, 000 is the full number of neurons in population, and Δ*t* = 0.1 *ms* is the integration step. We used the stochastic Heun method from the brian2 package for Monte Carlo simulations (Stimberg et al., 2019).

### 2.2. Synapse model

Synapses were simulated with the Tsodyks—Markram model (Tsodyks and Markram, 1997; Tsodyks et al., 1998; Moradi et al., 2022) as follows:


(3) $$\begin{array}{l} \frac{du}{dt}=-\frac{u}{\tau_{f}}+U_{inc}\cdot \left(1-u_{-}\right)\cdot w\cdot \nu_{pre}\cdot dt \end{array}$$

(4) $$\begin{array}{l} \frac{dx}{dt}=\frac{1-x-y}{\tau_{r}}-u_{+}x_{-}\cdot w\cdot \nu_{pre}\cdot dt \end{array}$$

(5) $$\begin{array}{l} \frac{dy}{dt}=-\frac{y}{\tau_{d}}+u_{+}x_{-}\cdot w\cdot \nu_{pre}\cdot dt \end{array}$$

*U*_*inc*_ is the increment of *u* produced by a spike. *u*_−_, *x*_−_ are variables just before the arrival of the spike, and *u*_+_ refers to the moment just after the spike. From the first equation (Equation 3), *u*_+_ = *u*_−_ + *U*_*inc*_·(1 − *u*_−_). Variables *y* and *x* mean the activated, deactivated, and recovered states, respectively, *u* is a fraction of resource ready for use. After each presynaptic spike, an instantaneous shift occurs from recovered to activated state. The amount of shift is determined by *u*. The active resources, then, decay to the deactivated state by the decay time constant τ_*d*_. Since synaptic resources are limited, the more resources stay in the deactivated state, the more a synapse is depressed. Synaptic resources exponentially recover from depression with the recovery time constant τ_*r*_. *w* is normalization coefficient, it makes sense of the density of connections. ν_*pre*_ is firing rate of presynaptic population. In Monte Carlo simulations ν_*pre*_ is defined by Equation 2, and in CBRD simulations, ν_*pre*_ is defined by Equation 9. For each connection parameters *U*_*inc*_, τ_*d*_, τ_*r*_, τ_*f*_, *w*, and *g*_*syn, max*_ are optimized. We consider infinitely large populations of presynaptic and postsynaptic neurons. In this context, variables mean the averages of all synapses between two populations [for detail, see Section 3 in Tsodyks et al. (1998)].

The synaptic current as follows:


(6) $$\begin{array}{l} I_{syn}=g_{syn,max}\cdot y\cdot \left(E_{syn}-V\right) \end{array}$$

where *g*_*syn, max*_ is maximal synaptic conductance and *E*_*syn*_ is reversal potential for synaptic current. The connection probability in Monte Carlo simulations is 0.5.

### 2.3. Conductance-based refractory density approach

Conductance-based refractory density (CBRD) approach simulates the distribution (ρ) of neurons in space-times after spike generation (t^*^) (Chizhov and Graham, 2007, 2008; Chizhov, 2017). The function ρ(t, t^*^) is described by the transfer equation as follows:


(7) $$\begin{array}{l} \frac{\partial \rho }{\partial t}+\frac{\partial \rho }{\partial t^{*}}=\rho H \end{array}$$

(8) $$\int_{0}^{\infty }\rho \cdot dt^{*}=1$$

The function *H* is defined as the probability of a single neuron to generate a spike when the mathematical expectation of the neuronal state variables is known (Equation 11).

Population firing rate is defined as follows:


(9) $$\nu (t)\equiv \rho (t,0)=\int_{0}^{\infty }\rho \cdot H\cdot dt^{*}$$

Population firing rate is determined by the probability of finding a neuron in the state of spike generation (t^*^ = 0).

The CBRD approach consider neurons only according to t^*^ variable. The state of each neuron is parameterized by this phase variable. For LIF neuron, its only state variable is *V*. Neuron model modified as follows:


(10) $$C_{m}(\frac{\partial V}{\partial t}+\frac{\partial V}{\partial t^{*}})=g_{L}(E_{L}-V)+I_{ext}+\sum I_{syn}$$

The function *H* has two components *A* and *B*:


(11) $$\begin{array}{l} H=\frac{A+B}{\tau_{M}} \end{array}$$

*A* is the probability for a neuron to cross the threshold because of noise, derived analytically in the study mentioned in Chizhov and Graham (2007) and approximated by exponential and polynomial functions for convenience. *B* is the probability of dischange of a neuron during depolarization due to external input, i.e., the hazard from drift in the voltage phase space. τ_*M*_ is time of membrane:


(12) $$\begin{array}{l} \tau_{M}=\frac{g_{tot}}{C_{m}} \end{array}$$

*C*_*m*_ is capacity of membrane, *g*_*tot*_ is total conductance of all channels, and it is sum of *g*_*L*_ and all synaptic conductances.


(13) $$\begin{array}{l} A=exp\left(0.0061-1.12T-0.257T^{2}-0.072T^{3}-0.0117T^{4}\right) \end{array}$$

(14) $$\begin{array}{l} B=-\sqrt{2}\cdot {\left[\frac{dT}{dt}\right]}_{+}\cdot F_{T}\cdot \tau_{M} \end{array}$$

[*z*]_+_ is heaviside function:


(15) $${\left[z\right]}_{+}:=\{\begin{array}{ll} 0 & \text{if }z\leq 0;\\ 1 & \text{if }z>1 \end{array}$$

(16) $$\begin{array}{l} T=\frac{V^{T}-V}{\sqrt{2}\sigma_{m}} \end{array}$$

where *V* is membrane potential, σ_*m*_ is the noise amplitude, and *V*^*T*^ is the threshold for spike generation. *T* is the membrane potential relative to the threshold, scaled by noise amplitude.


(17) $$\begin{array}{l} \frac{dT}{dt}=\frac{-1}{\sqrt{2}\sigma_{m}}\frac{dV}{dt} \end{array}$$

(18) $$\begin{array}{l} F_{T}=\sqrt{2/\pi }\cdot exp\left(-\left(T^{2}\right)\right)/\left(1+erf\left(T\right)\right) \end{array}$$

where *erf* is Gauss error function. The function *F*_*T*_ characterizes the firing probability in the regime of fast changes of *T*.

All values for model are same as for Monte Carlo simulations.

### 2.4. Numerical methods for CBRD simulations

In general, the equations can be written as follows:


(19) $$\begin{array}{l} \frac{\partial z}{\partial t}+\frac{\partial z}{\partial t^{*}}=S_{z}\left(t,t^{*}\right) \end{array}$$

where *z*(t, t^*^) is one of the functions ρ or *V*, and $S_{z}\left(t,t^{*}\right)$ is the source term of the equation.

Numerical scheme was taken from Harten and Osher (1987). Numerical scheme is as follows:


(20) $$\begin{array}{l} z_{i}^{n+1}=z_{i}^{n}-\frac{\text{Δ}t}{\text{Δ}t^{*}}\left(z_{i}^{n}-z_{i-1}^{n}+\frac{1}{2}\left(1-\frac{\text{Δ}t}{\text{Δ}t^{*}}\right)\left(w_{i}^{z}-w_{i-1}^{z}\right)\right)+\text{Δ}t\cdot {\left(S_{z}\right)}_{i}^{n} \end{array}$$

*n* is index in time, and *i* is index in space.


(21) $$\begin{array}{l} w_{i}^{z}=limiter\left(z_{i+1}-z_{i},z_{i}-z_{i-1}\right) \end{array}$$

(22) $$limiter(a,b)=\{\begin{array}{ll} 0 & \text{if }ab<=0;\\ -min(0.5\text{ |}ab\text{|},2\;min(\text{|}a\text{|},\text{|}b\text{|})) & \text{if }a<0,\;ab>0;\\ min(0.5\text{ |}ab\text{|},2\;min(\text{|}a\text{|},\text{|}b\text{|})) & \text{otherwise} \end{array}$$

#### 2.4.1. Bound conditions

Bound values $w_{0}^{z}=0$, $w_{N}^{z}=0$. Firing rate (left bound for ρ) is calculated as follows:


(23) $$\begin{array}{l} \rho_{0}^{n+1}=\rho_{0}^{n}-\frac{\text{Δ}t}{\text{Δ}t^{*}}\rho_{0}^{n}-\text{Δ}t\overset{N}{\underset{i=0}{\sum }}{\left(S_{\rho }\right)}_{i}^{n} \end{array}$$

Left bound for *V*:


(24) $$\begin{array}{l} V_{0}^{n+1}=V_{reset} \end{array}$$

Right bound for ρ:


(25) $$\rho_{N}^{n+1}=\rho_{N}^{n}+\frac{\text{Δ}t}{\text{Δ}t^{*}}(\rho_{N-1}^{n}+\frac{1}{2}(1-\frac{\text{Δ}t}{\text{Δ}t^{*}})w_{N-1}^{\rho })+\text{Δ}t\cdot {\left(S_{\rho }\right)}_{N}^{n}$$

Right bound for *V*:


(26) $$\begin{array}{l} V_{N}^{n+1}=V_{N}^{n}+\text{Δ}t{\left(S_{V}\right)}_{N}^{n} \end{array}$$

Δ*t* = 0.1 *ms*, Δt^*^ = 0.5 *ms*, and *N* = 400 is the number of spatial states. All simulations using the CBRD approach were performed with the TensorFlow package (version 2.10.0). All gradients were calculated using automatic differentiation (Abadi et al., 2015).

### 2.5. Target firing rates, inputs, and loss function

Firing rate of inputs and target firing rate for simulated populations as follows:


(27) $$\begin{array}{l} FR_{pop}\left(t\right)=\frac{FR_{mean,pop}}{I_{0}\left(\kappa_{pop}\right)}\cdot exp\left(\kappa_{pop}\cdot \left(cos\left(2\pi \omega_{\theta }t-\phi_{pop}\right)\right)\right) \end{array}$$

where *FR*_*pop*_(*t*) is population firing rate in time in spikes/second. *FR*_*mean,pop*_ is mean population firing rate (spikes/second). κ_*pop*_ is a measure of concentration of von Mises distribution, *I*_0_(κ) is a zero order Bessel function. ω_θ_ = 7 *Hz* is frequency of theta rhythm, and ϕ_*pop*_ is peak phase of population firing. Parameters for all populations are presented in Table 1. Authors of experimental studies usually use R (ray length) as a measure of phase variation rather than κ (Mardia and Jupp, 1999). κ is calculated from *R* with following approximation (Mardia and Jupp, 1999):


(28) $$\kappa (R)=\{\begin{array}{ll} 2\cdot R+R^{3}+5/6\cdot R^{5} & \text{if }R<0.53;\\ -0.4+1.39\cdot R+0.43/(1-R) & \text{if }0.53\leq R<0.85;\\ 1/(3\cdot R-4\cdot R^{2}+R^{3}) & \text{if }R\geq 0.85 \end{array}$$

**Table 1:** Parameters of the target function.

**Population**	***R***	**ω_θ_ (Hz)**	***FR*_*mean*_ (spike/second)**	**ϕ (rad)**	**Sources**
ca3pyr	0.3	7.0	0.5	1.58	Mizuseki et al., 2009
ca1pyr	0.2	7.0	0.5	3.14	Mizuseki et al., 2009
ec3	0.2	7.0	1.5	-1.57	Mizuseki et al., 2009
pvbas	0.3	7.0	24	1.57	Lapray et al., 2012, Varga et al., 2012, Katona et al., 2014, Somogyi et al., 2014
olm	0.3	7.0	30	3.14	Varga et al., 2012, Katona et al., 2014, Somogyi et al., 2014
cckbas	0.3	7.0	9.0	-1.57	Klausberger et al., 2005, Somogyi et al., 2014
bis	0.3	7.0	27.0	3.14	Katona et al., 2014, Somogyi et al., 2014
aac	0.3	7.0	29.0	0.0	Somogyi et al., 2014, Varga et al., 2014
ivy	0.3	7.0	4.0	-1.57	Lapray et al., 2012, Varga et al., 2012, Somogyi et al., 2014
ngf	0.3	7.0	8.0	0.0	Sakalar et al., 2022

Artificial inputs in Monte Carlo simulations are modeled as Poisson generators with rate given by the Equation 27.

The loss function for estimating the discrepancy between the simulation and the target function as follows:


(29) $$\begin{array}{l} L_{simulation}=\overset{K}{\underset{k=1}{\sum }}\overset{T}{\underset{t_{n}=0}{\sum }}{\left(ln\left(\rho_{target}\left(t_{n}\right)+1\right)-ln\left(\rho_{simulated}\left(t_{n}\right)+1\right)\right)}^{2} \end{array}$$

Summation is carried out by time and all populations of neurons. *K* = 7 is the number of populations in the model. *T* is the number of time steps in the simulation.

The optimized parameters must be within certain bounds. In particular, all parameters of synapses should be positive, and *U*_*inc*_ should not exceed one. We have added barrier terms to the loss function.


(30) $$\begin{array}{l} L_{barrier}=\sum_{m=1}^{M}\left(\text{−}0.001\cdot ln(100g_{syn,max})\right)+\\ \;+\sum_{m=1}^{M}\left(\text{−}0.001\cdot ln(100\tau_{r})\right)+\\ \;+\sum_{m=1}^{M}\left(\text{−}0.001\cdot ln(100\tau_{f})\right)+\\ \;+\sum_{m=1}^{M}\left(\text{−}0.001\cdot ln(100\tau_{d})\right)+\\ \;+\sum_{m=1}^{M}\left(\text{−}0.001\cdot ln(100U_{inc})\right)+\\ \;+\sum_{m=1}^{M}\left(\text{−}0.001\cdot ln(100w)\right)+\\ \;+\sum_{m=1}^{M}\left(\text{−}0.001\cdot ln(100(1-U_{inc})\right) \end{array}$$

Summarization is carried out for all synapses in the model. M = 49 M is the number of connections in the model.

Full loss function for optimization as follows:


(31) $$\begin{array}{l} L_{full}=L_{simulation}+L_{barrier} \end{array}$$

We have used Adam optimizer with standard parameters: learning rate = 0.001, *β*_1_ = 0.9, and *β*_2_ = 0.999 (Kingma and Ba, 2014).

## 3. Results

### 3.1. Optimization of the model

We considered a network of biologically plausible networks consisting of seven populations of interneurons. The graph of connections is complex and contains 49 connections (Figure 1) (Moradi et al., 2022). Excitatory inputs were not modeled as neural populations but were taken into account artificial generators (functions from time). Each synapse is described by six parameters (Equations 3–5). We fit all these parameters. In addition, the *I*_*ext*_ parameter for each neuron population was fit in simulations (Equation 1). Thus, we needed to tune 301 model parameters. Our approach contains elements of novelty, so we will begin the description of the results with a brief description of the formulation and solution of the optimization problem.

We propose to fit the parameters by reducing the process of building a model to solving the optimization problem. In other words, it is proposed to find the optimal parameters of the model so that it describes the experimental data in the best way. The requirement to be able to configure numerous parameters makes it impossible to use zero-order optimization methods. Gradient estimation is possible by using automatic differentiation packages (Tensorflow, Torch) (Abadi et al., 2015) or the adjoint state method (Chen et al., 2019; Sun et al., 2020). However, these approaches require direct computation of variables by which differentiation will be carried out.

The activity of neurons in the real brain is noisy. The vast majority of experimental phenomena are obtained as a result of averaging the activity of one or more neurons depending on external stimuli or internal state. Modulation of neuronal activity by theta rhythm is just one example. In other words, the experimental phenomena of neural network dynamics are empirical distributions. Modeling consists of describing the parameters of distributions. In this context, direct simulation of distributions is necessary to be able to estimate gradients.

We have used the population approach for modeling, since it allows us to describe the population firing rate. As a population approach, we chose the CBRD (conductance-based refectory density) method (Chizhov and Graham, 2007, 2008; Chizhov, 2017). Direct simulation of the firing rate makes it possible to estimate the gradient of the loss function from the model parameters. All operators of the numerical scheme of the CBRD model are differentiable, so automatic differentiation can be applied (Section 2.4). Estimating the gradient of the loss function makes it possible to apply gradient descent methods to optimize model parameters (Equation 31). All simulations using the CBRD approach were performed with the TensorFlow package, and gradients were calculated using automatic differentiation (Abadi et al., 2015). As initial conditions for optimization, we used the average values of the parameters described in the study by Moradi et al. (2022). We ran Monte Carlo simulations with the parameters found using optimization. Monte Carlo simulations were used to control of the accuracy of the CBRD approach.

We have introduced a target activity function for each population (Equation 27). This function approximate experimental data about modulation of neuronal activity by the theta rhythm. It describes the population firing rate of each group of neurons over time. The target function takes into account the average firing rate and theta rhythm modulation. Theta rhythm modulation is determined by the mean phase (circular average of the phases of neuronal discharges) and the phase variation (ray length - R). The population firing rate of excitatory inputs was described by similarly functions (Equation 27).

This approach has advantages and disadvantages. On the one hand, population models are only applicable to simple models of neurons. In our study, we used the LIF (leaky integrate-and-fire) model (Equation 1). On the other hand, the use of gradient descent allows optimization over a much larger number of parameters than gradient-free methods.

### 3.2. Description of the model results

**Figure 2:**
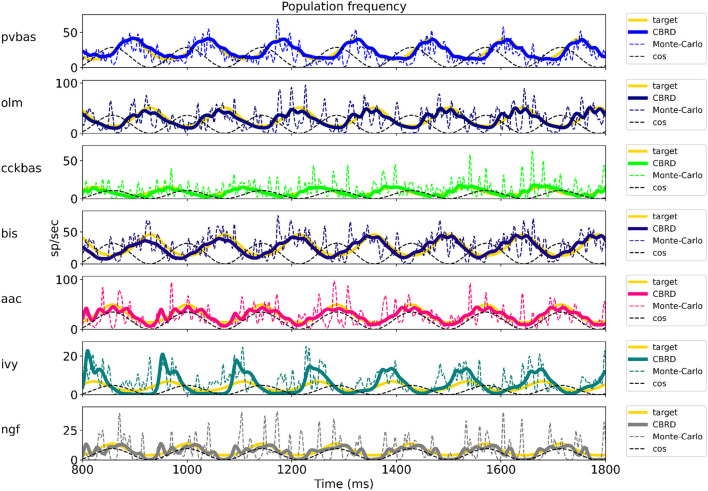
The results of model optimization. For each population, plots show the target function, and the population spike rate obtained with the CBRD approach using Monte Carlo simulation. In total, 1 second of simulation is shown after stabilization of the model dynamic mode. The notation of neurons is similar to Figure 1.

**Figure 3:**
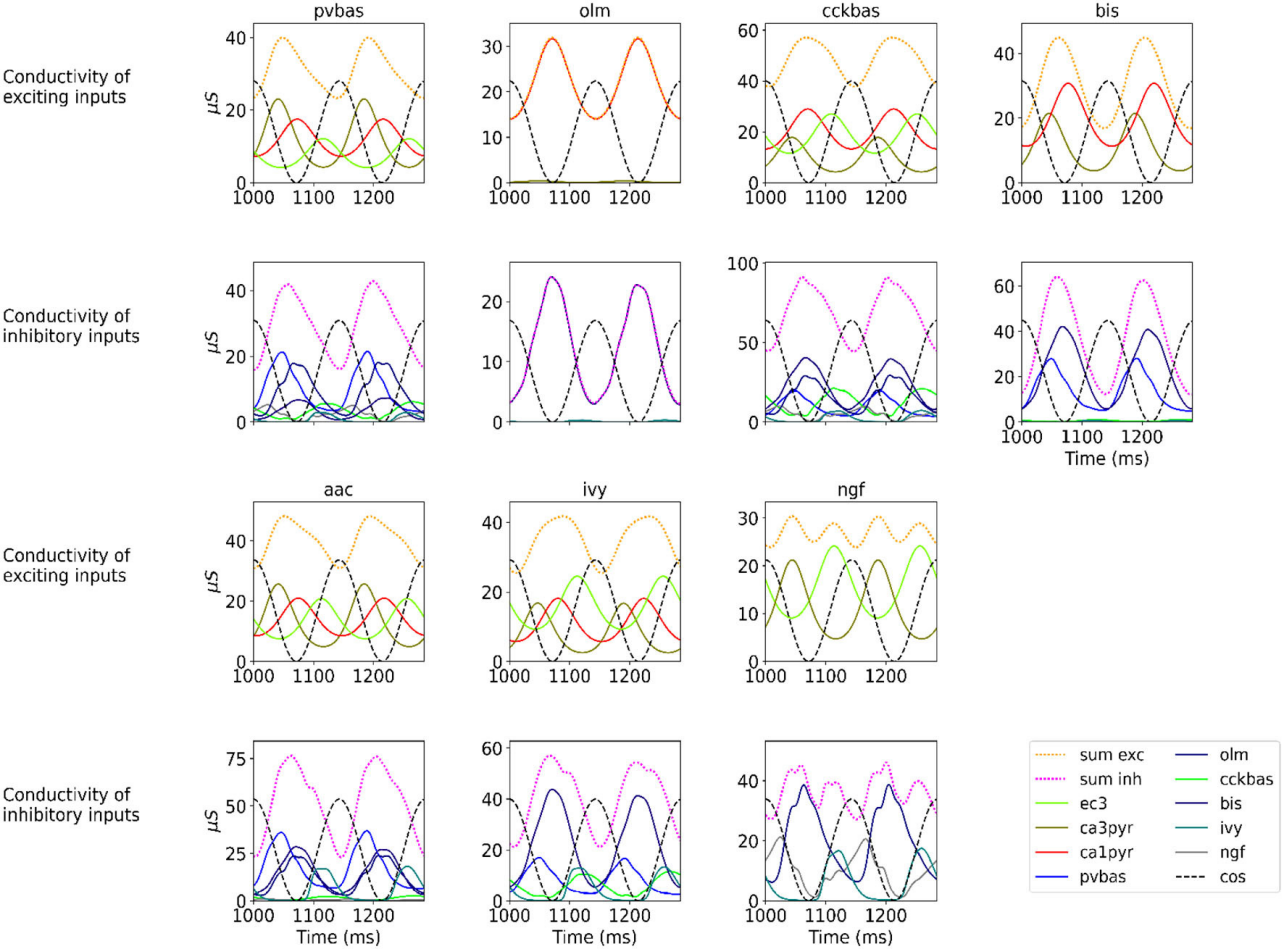
Synaptic conductivities. Two theta cycles of the simulation using the CBRD approach are shown. Excitatory (**upper** series of plots) and inhibitory (**lower** series of plots) conductivities are shown for each population. The color indicates presynaptic populations. Each plot shows the sum of conductivities, “sum exc" and “sum inh" notes sum of excitatory and inhibitory conductivities, respectively. The notation of neurons is similar to Figure 1.

Figure 2 shows the optimization results. For each population, the target function, the results of simulation using the CBRD approach, and the results of Monte Carlo simulation with optimal parameters are presented. The model under consideration can approximate the target functions for all populations of interneurons with significant accuracy. The simulation results are in the confidence intervals of experimental estimates. The model describes all the required characteristics of the experimental data: the average spike rate, the average phase of the theta rhythm, and the ray length (R). We also note a good agreement between the results of Monte Carlo simulations and simulations using the CBRD approach. In the next step, we looked at how the model works. Figure 3 shows the dynamics of synaptic conductivities in each neuron population. Both excitatory and inhibitory conductivities have an unimodal distribution over the theta cycle for all neuron populations, except neurogliaform cells. An unexpected result is that the peaks of the excitatory and inhibitory inputs coincide. This effect is observed for all populations of neurons. The ratio of the total excitatory to inhibitory conductivity remains approximately constant over the theta cycle and ranges from 0.7 to 2.4 for different populations. Since the reversion potential for AMPA receptors (0 mV) is much further from the resting potential than the reversion potential of GABA-A receptors (–75 mV). Excitatory currents dominate over inhibitory ones and determine the dynamics of neuronal discharges. For all neuron populations except axo-axonal ones, the peak of excitatory input is located near the peak of firing rate. The dynamics of axo-axonal neurons in the model is determined by the external current (Supplementary Table S1). All optimal parameters are given in Supplementary material.

We performed optimization for a similar network with synapses without short-term plasticity. The results are presented in the Supplementary material. For a part of neural populations (pvbas, olm, bis, and aac), it fits the parameters to describe phase relations. However, the remaining populations are completely silent (ivy, ngf, and cckbas). These results show that short-term plasticity is a necessary element of the model for reproducing phase relations.

### 3.3. Generalizing power of the model

**Figure 4:**
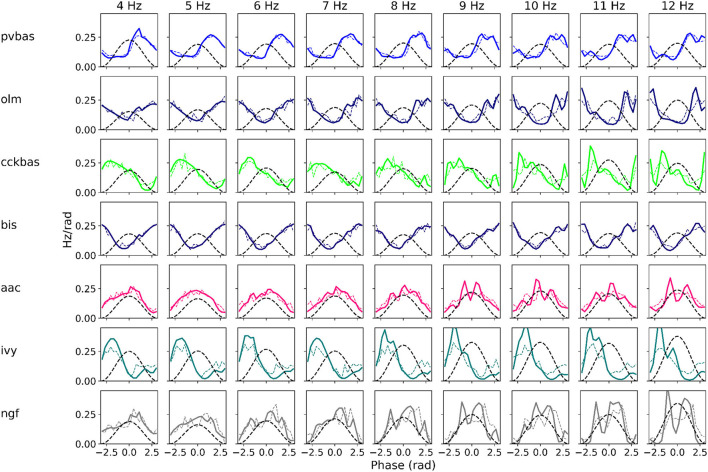
Phase relations. Each plot shows the distribution of activity of neuronal populations by theta rhythm phase. The frequency of the theta rhythm was set in the equations of the exciting inputs. All simulations were carried out with optimal parameters found at a theta rhythm frequency of 7 Hz. Simulations were carried out using the CBRD approach (thick lines) and Monte Carlo (dashed line). The notation of neurons is similar to Figure 1.

**Figure 5:**
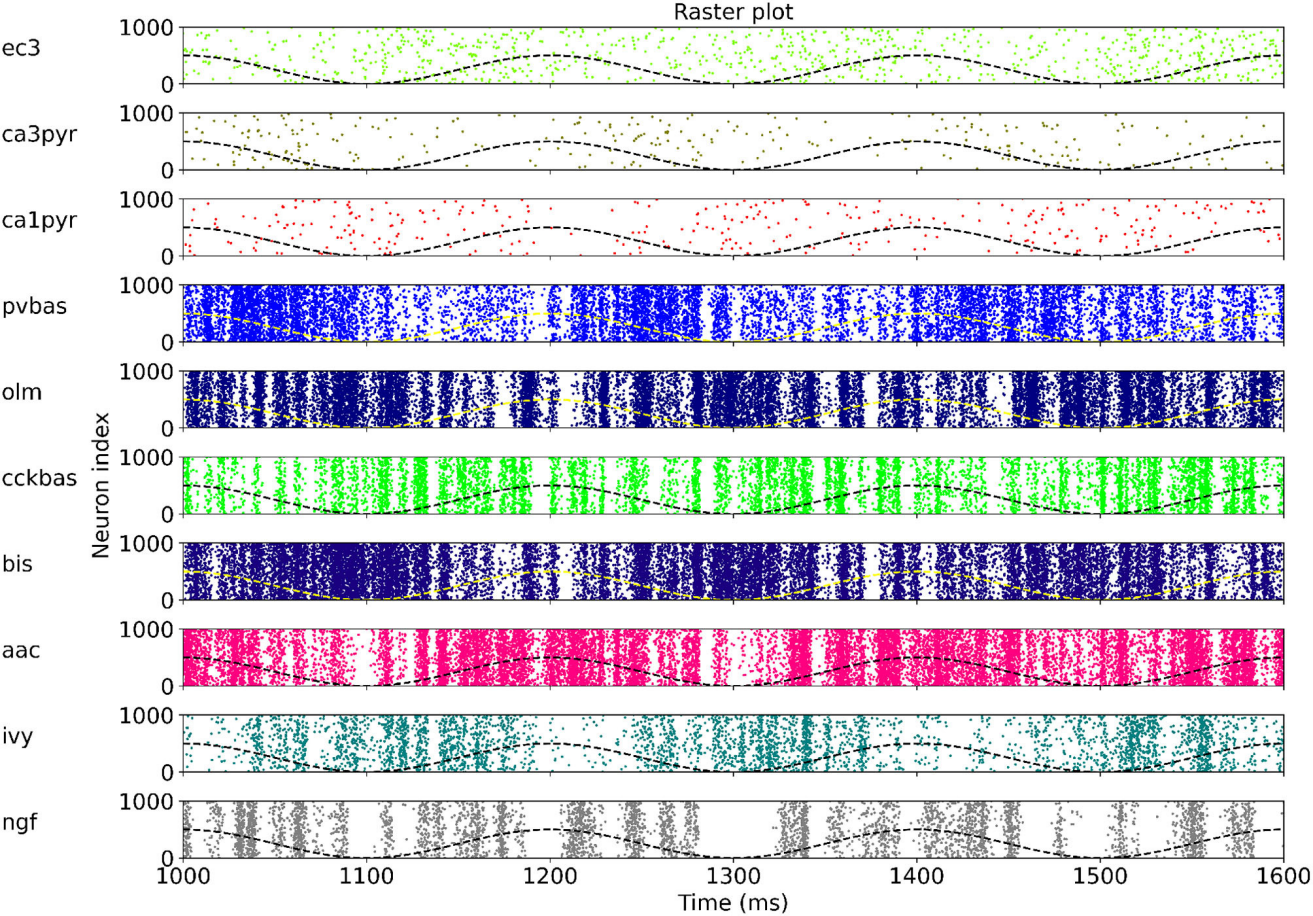
Raster plots for Monte Carlo simulation with the theta rhythm frequency of 5 Hz. For each population, only 1,000 neurons are shown, but 4,000 neurons are simulated. The dashed line is reference cos. The notation of neurons is similar to Figure 1.

At the last stage of research, we tested the stability of the found solution at other theta rhythm frequencies. We searched for optimal parameters at a theta rhythm frequency of 7 Hz. Figure 4 shows the results of simulations with varying theta rhythm frequency. Figure 5 shows raster plots for a Monte Carlo simulation with a theta rhythm frequency of 5 Hz. In this series of computational experiments, we changed the frequencies of the input populations. The model parameters were used that were found earlier. Over the entire frequency range (4–12 Hz), the shape of the distribution of neuronal activity over the theta rhythm phase is preserved. The theta rhythm frequency is a parameter that varies greatly in animal experiments, for example, it correlates with the animal's running speed (Hinman et al., 2011; Ledberg and Robbe, 2011; Long et al., 2014; Justus et al., 2017). The conservation of the phase relations of interneuron activity with a change in the theta rhythm frequency is an important property of our model. These experiments can be considered as analogous to cross-validation used in machine learning. In the language of machine learning, we can say that our model shows good quality on the “test set”, i.e., samples that did not participate in the “training”.

## 4. Discussion

### 4.1. Assumptions and limitations of the model

Our results are based on several key assumptions. We used the von Mises function to describe the inputs and target activities for interneurons (Equation 27). These assumptions of the model are based on experimental data that more than 85% of pyramidal neurons in the CA3 and CA1, three fields of the EC layer are modulated by theta rhythm (Mizuseki et al., 2009). The proportion of interneurons of the CA1 field modulated by the theta rhythm is 95% (Mizuseki et al., 2009). The degree of phase modulation R for all hippocampal neurons is in the range of 0.2–0.4; we used R = 0.3 as the mean estimate. Binding phases for exciting inputs and local interneurons of the CA1 field (Table 1) have been measured in several experimental studies and are reliable (Klausberger et al., 2003; Somogyi and Klausberger, 2005; Mizuseki et al., 2009; Somogyi et al., 2014). Our model takes into account the average discharge frequencies of input populations and interneurons (Table 1). We selected the values for simulations as the mean and median values measured in the literature. The exact values of the mean firing rate are not important in our context, since the discharge frequency of the presynaptic population is linearly related to the weight of the synaptic connection with the postsynaptic population. In other words, a decrease in the firing rate of the presynaptic population can be compensated by an increase in synaptic conduction in postsynaptic neurons. For example, if a neuron receives inputs from *N* neurons with a connection density *p*, there are *N*·*p* synapses on it. If *N* is decreased but *p* is increased by the same number of times, the number of synapses will not change. There is a coefficient *w* in the synapse model (Equations 3–5). It takes into account the density of connections. We have optimized the coefficient *w*, so the density of connections is indirectly taken into account in the model. Thus, our model results describe the network of the CA1 field up to normalization.

We used a homogeneous representation of each population of interneurons. Recent studies show that interneurons are involved in the formation of neural ensembles, i.e., their activity is modulated by external stimuli (Geiller et al., 2022). However, a study of simultaneous registration of a large number of interneurons of different classes shows that cells of all classes are active simultaneously for times of hundreds of milliseconds (Geiller et al., 2020). Thus, it is reasonable to assume that a significant proportion of neurons in each population is active at the same time. Notably, the effect of simultaneous activity of interneurons of different populations is observed, despite the inhibitory connections of populations between each other (Bezaire and Soltesz, 2013). Our model reproduces this effect well. Due to the homogeneity of each population, we used the same number of neurons in direct simulations. Interneurons are unevenly distributed across classes. For example, population of Ivy neurons are six times larger than axo-axonal cells (Bezaire and Soltesz, 2013). A representative representation of neurons would not improve the accuracy of the model, but it would increase computational costs. We did not take into account the input to the model from the medial septum. Recent studies show that the strongest rhythmic input from the septum goes to the interneurons of the CA3 field and the entorhinal cortex (Joshi et al., 2017; Unal et al., 2018; Viney et al., 2018). Other data demonstrate the importance of input from the CA3 field and the entorhinal cortex to the CA1 field for theta rhythm in it (López-Madrona et al., 2020; Zutshi et al., 2022). Together, these results lead to the hypothesis that the theta rhythm in the CA1 field results from secondary rhythmic inputs rather than direct input from the medial septum. There are two significant limitations of our model. The first is usage of the LIF model of neurons. Most types of interneurons have slow potassium channels (Bezaire et al., 2016; Komendantov et al., 2019), which can make a significant contribution to the frequency of neuronal discharges due to spike-frequency adaptation. OLM neurons have pronounced H-currents (Saraga et al., 2003), which can also make a significant contribution to the impulsing of these neurons due to depolarization or resonant properties (Avella Gonzalez et al., 2015). The usage of the LIF model is dictated by a limitation of the population approach. We did not explicitly simulate pyramidal neurons of the CA1 field due to the limitations of the population method. Different classes of interneurons have entrances to different compartments of pyramidal cells. The correct inclusion of pyramid neurons in the network requires the use of multicompartment models to describe them. The problem of finding optimal synaptic inputs to pyramidal neurons can be solved separately.

The second limitation is that we did not take into account all populations of interneurons. This limitation is based on the weak knowledge of other populations of interneurons. The lack of data makes it impossible to include other populations in the model. Accounting for more populations can change the balance of inhibition and shift its peak.

### 4.2. Comparison with other models

The literature presents several attempts to explain the phase relations between neurons and theta rhythm. The key idea of the research is to determine the optimal structure of the input from the medial septum and excitatory inputs (Cutsuridis et al., 2010; Cutsuridis and Hasselmo, 2012; Cutsuridis and Poirazi, 2015; Bezaire et al., 2016; Mysin et al., 2019; Mysin, 2021). Our results do not negate the contribution of external inputs but rather dismantle an additional mechanism for stabilizing phase relations. Models with a few interneuron populations give a good approximation of the distribution of neurons in theta rhythm phases due to input from the medial septum (Cutsuridis et al., 2010; Mysin et al., 2019). However, with an increase in the number of interneuron classes in models, the quality of the approximation of phase relations decreases (Cutsuridis and Poirazi, 2015; Bezaire et al., 2016; Mysin, 2021). Although in the cited studies, the authors used a different input structure. We believe that taking into account the detailed structure of the inputs to the CA1 field in the model is not sufficient to explain the phase relations of interneurons. In this study, we have shown that the short-term plasticity of synapses between populations of interneurons may be the missing component in the stabilization of phase relationships.

### 4.3. Comparison of simulation results with experimental data

The main result of our study is an unimodal distribution of excitation and inhibition with coinciding peaks for most types of interneurons. Checking this fact is a complex experimental task. In the literature, there is a study of synaptic currents on PV basket and OLM neurons during theta rhythm generation in hippocampal slices (Huh et al., 2016). The authors also found that unimodal excitation and inhibition coincided in time. The peak of inhibition occurred 12 ms after the peak of excitation for PV basket neurons and 7 ms after the peak of arousal for OLM neurons (Huh et al., 2016). We used experimental data *in vivo* as the basis for our model; therefore, comparison with data on hippocampal preparations is limited.

### 4.4. Prospects for applying population model optimization

Several attempts have been made in the literature to optimize networks of spiking neurons (Lee et al., 2016; Wunderlich and Pehle, 2021; Zenke and Vogels, 2021; Ramezanian-Panahi et al., 2022). However, these studies are aimed at finding practical applications in the field of data analysis not brain modeling. These approaches are not directly applicable for building a model in neuroscience. The activity of real individual neurons is noisy. The vast majority of experimental phenomena are obtained as a result of averaging the activity of one or several neurons, depending on external stimuli or internal state. The phase relations of neuronal activity are one of the many examples. Another example is place cells. This phenomenon can be described as the distribution of neuronal activity depending on the position of the animal. Phase precession of place cells is the distribution of neuronal activity depending on the position of the animal and the phase of the theta rhythm. Population models describe physiological phenomena in the language of distributions. Modeling complex systems require numerous equations and parameters. Projects such as the Human Brain Project or the Hippocampus Project aim to model the brain by collecting and organizing parameters for equations (Wheeler et al., 2015; Bjerke et al., 2018). Optimization of population models can well complement the processes of collecting parameters. The development of the mathematical apparatus of population models may be one of the reasons for a breakthrough in the construction of large-scale models of the brain.

### 4.5. General remarks

Our model shows a possible mechanism for the formation of phase relations between interneurons and theta rhythm in the CA1 field. We assume that a similar mechanism operates in other areas of the hippocampal formation. The establishment of phase relationships among interneurons leads to the coupling of principal neurons to the phase of the theta rhythm. Rhythmic inputs to the principal neurons creates oscillations of local field potential (Buzsáki et al., 2012; Einevoll et al., 2013). This, in turn, synchronizes the regions of the hippocampus with each other through the projections of principal neurons, which is necessary for the transmission of information (Bastos et al., 2015; Mysin and Shubina, 2022). Synchronization of hippocampal regions creates a capability for the formation of phase precession (Burgess and O'Keefe, 2011; Fernández-Ruiz et al., 2017; Zutshi et al., 2022). Another result of the synchronization of different areas of the hippocampus is the emergence of coupling of theta and gamma rhythms. For example, a slow gamma rhythm is formed in a feedback loop between PV basket and principal neurons (Colgin and Moser, 2010; Buzsáki and Wang, 2012). In the CA1 field, excitation from the CA3 field is necessary for the formation of a slow gamma rhythm (Colgin, 2015; Fernández-Ruiz et al., 2017). PV basket neurons CA1 have a peak of discharges in the descending phase of theta rhythm, and the input from field CA3 falls into the same phase (Mizuseki et al., 2009; Belluscio et al., 2012). Therefore, a model that reproduces phase relationships with respect to the theta rhythm will have a good predictive ability with respect to other phenomena associated with the theta rhythm. Identification of the mechanisms of formation of the dynamics of the activity of interneurons during the generation of the theta rhythm is important for understanding the processing of information and the formation of hippocampal rhythms.

## Data availability statement

Publicly available datasets were analyzed in this study. This data can be found at: https://static-content.springer.com/esm/art%3A10.1038%2Fs42003-022-03329-5/MediaObjects/42003_2022_3329_MOESM3_ESM.xlsx.

## Author contributions

IM: main idea, coding, and article writing.
