# Supplementary material for: Phase relations of interneuronal activity relative to theta rhythm
Source: Front Neural Circuits. 2023 Jul 6;17:1198573. doi: 10.3389/fncir.2023.1198573 (PMC10358363; doi:10.3389/fncir.2023.1198573)
Supplement: Supplementary file 1 [file Data_Sheet_1.PDF]

Supplementary Material

1 OPTIMIZATION RESULTS OF THE MODEL WITH NONPLASTIC SYNAPSES

Nonplastic synapses simulated with equation:

$$\tau_1 \cdot \tau_2 \cdot \frac{d^2 g_s}{dt^2} + (\tau_1 + \tau_2)\frac{dg_s}{dt} = g_s \cdot w \cdot \nu_{pre} \tag{S1}$$

The synaptic current:

$$I_{syn} = g_{syn,max} \cdot g_s \cdot (E_{syn} - V) \tag{S2}$$

$g_{syn,max}, \tau_1, \tau_2, w$ were optimized. The barrier term in the loss function has been modified:

$$\begin{aligned} L_{barrier} = &\sum_{m=1}^{M} (-0.001 \cdot ln(100 g_{syn,max})) + \\ &+ \sum_{m=1}^{M} (-0.001 \cdot ln(100\tau_1)) + \\ &+ \sum_{m=1}^{M} (-0.001 \cdot ln(100\tau_2)) + \\ &+ \sum_{m=1}^{M} (-0.001 \cdot ln(100w)) \end{aligned} \tag{S3}$$

Summarization is carried out for all synapses in the model.

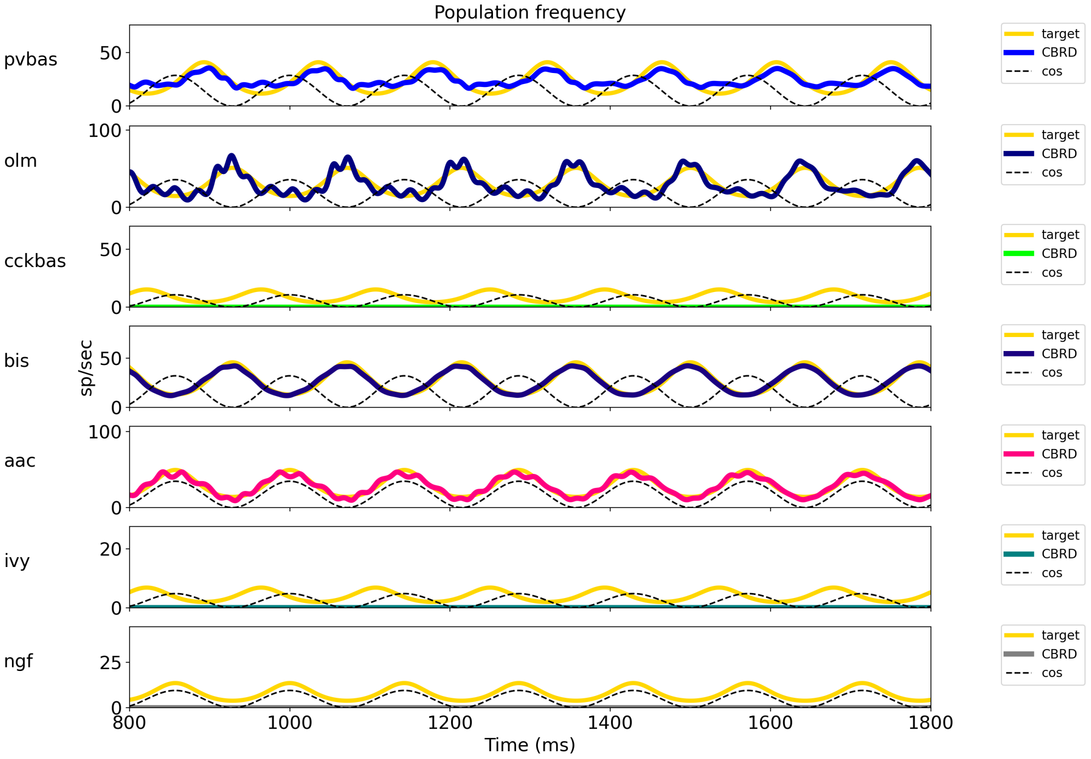

Figure S1. Optimization results of the model with nonplastic synapses. For each population, plots show the target function, the population spike rate obtained with the CBRD approach. One second of simulation is shown after stabilization of the model dynamic mode. The notation of neurons is similar to Fig. 1 of the article.

2 SUPPLEMENTARY TABLES

Optimal parameters for simulations with plastic synapses.

Table S1. $I_{ext}, \mu A/cm^2$

	pvbas	olm	cckbas	bis	aac	ivy	ngf
Iext	0.12	0.26	-0.06	0.33	0.2	-0.06	0.3

Table S2. $g_{syn,max}$ mS

	Postsynaptic						
Presynaptic	pvbas	olm	cckbas	bis	aac	ivy	ngf
ca3pyr	1.14	0.68	1.14	1.14	1.16	1.16	1.63
ca1pyr	1.47	1.82	1.83	2.22	1.61	2.08	-
ec3	1.38	-	1.9	-	1.61	1.73	1.77
pvbas	3.26	-	3.09	2.88	3.58	3.23	-
olm	1.4	-	1.84	-	1.69	-	1.83
cckbas	1.13	-	1.48	1.42	1.3	1.62	-
bis	1.39	1.16	1.69	1.61	1.43	1.64	-
aac	-	-	-	-	-	-	-
ivy	1.48	1.33	1.6	1.46	1.65	1.62	1.61
ngf	1.42	-	1.64	-	1.45	-	1.64

Table S3. w

	Postsynaptic						
Presynaptic	pvbas	olm	cckbas	bis	aac	ivy	ngf
ca3pyr	6.57	4.26	4.41	4.91	6.38	4.6	5.02
ca1pyr	6.3	5.83	4.65	5.15	5.63	4.33	-
ec3	1.63	-	1.77	-	1.85	1.82	1.89
pvbas	0.09	-	0.06	0.1	0.14	0.05	-
olm	0.12	-	0.12	-	0.11	-	0.23
cckbas	0.06	-	0.15	0.02	0.03	0.1	-
bis	0.04	0.15	0.16	0.2	0.11	0.15	-
aac	-	-	-	-	-	-	-
ivy	0.08	0.03	0.09	0.03	0.18	0.16	0.15
ngf	0.1	-	0.11	-	0.04	-	0.17

Table S4. τ_d, ms

	Postsynaptic						
Presynaptic	pvbas	olm	cckbas	bis	aac	ivy	ngf
ca3pyr	5.61	5.34	4.78	6.14	5.61	6.51	4.18
ca1pyr	3.25	3.21	2.74	3.45	3.4	3.82	-
ec3	3.89	-	3.5	-	3.8	4.27	4.34
pvbas	3.79	-	4.19	5.07	4.06	5.04	-
olm	5.63	-	5.52	-	5.89	-	6.99
cckbas	7.01	-	6.69	8.18	7.39	8.41	-
bis	7.46	7.93	8.03	10.21	8.01	10.68	-
aac	-	-	-	-	-	-	-
ivy	6.57	7.34	7.01	9.03	7.05	9.37	8.86
ngf	6.5	-	6.25	-	6.64	-	8.84

Table S5. τ_f, ms

	Postsynaptic						
Presynaptic	pvbas	olm	cckbas	bis	aac	ivy	ngf
ca3pyr	29.78	38.38	61.44	27.59	31.54	21.98	50.32
ca1pyr	76.9	106.95	200.09	45.85	68.72	29.95	-
ec3	38.31	-	97.25	-	43.02	50.31	50.31
pvbas	15.09	-	27.37	21.41	17.51	17.37	-
olm	16.62	-	36.47	-	19.45	-	20.7
cckbas	53.25	-	89.24	49.12	68.12	39.24	-
bis	12.43	17.63	40.15	14.36	15.79	11.64	-
aac	-	-	-	-	-	-	-
ivy	14.36	20.69	35.03	16.88	18.31	13.43	25.56
ngf	20.48	-	40.38	-	23.47	-	25.58

Table S6. τ_r, ms

	Postsynaptic						
Presynaptic	pvbas	olm	cckbas	bis	aac	ivy	ngf
ca3pyr	440.07	358.57	330.99	369.46	388.4	419.17	345.25
ca1pyr	327.26	201.9	170.37	242.43	295.47	294.63	-
ec3	363.56	-	274.88	-	331.47	345.21	345.24
pvbas	635.7	-	576.53	584.65	596.75	598.76	-
olm	650.22	-	527.62	-	602.4	-	578.02
cckbas	752.48	-	638.85	628.55	663.69	653.68	-
bis	776.48	717.22	546.21	710.0	738.36	760.08	-
aac	-	-	-	-	-	-	-
ivy	736.06	669.69	569.35	641.27	679.4	688.89	553.77
ngf	598.63	-	500.4	-	547.04	-	553.66

Table S7. U_{inc}

	Postsynaptic						
Presynaptic	pvbas	olm	cckbas	bis	aac	ivy	ngf
ca3pyr	0.37	0.0	0.17	0.22	0.38	0.16	0.14
ca1pyr	0.25	0.32	0.25	0.29	0.27	0.18	-
ec3	0.14	-	0.28	-	0.3	0.3	0.21
pvbas	0.29	-	0.27	0.2	0.3	0.31	-
olm	0.29	-	0.31	-	0.29	-	0.36
cckbas	0.34	-	0.26	0.23	0.34	0.2	-
bis	0.22	0.26	0.35	0.35	0.32	0.34	-
aac	-	-	-	-	-	-	-
ivy	0.29	0.19	0.32	0.23	0.38	0.28	0.31
ngf	0.29	-	0.27	-	0.21	-	0.28
